# A new paradigm considering multicellular adhesion, repulsion and attraction represent diverse cellular tile patterns

**DOI:** 10.1371/journal.pcbi.1011909

**Published:** 2025-04-21

**Authors:** José A. Carrillo, Hideki Murakawa, Makoto Sato, Miaoxing Wang

**Affiliations:** 1 Mathematical Institute, University of Oxford, Oxford, United Kingdom; 2 Faculty of Advanced Science and Technology, Ryukoku University, Otsu, Shiga, Japan; 3 Mathematical Neuroscience Unit, Institute for Frontier Science Initiative, Laboratory of Developmental Neurobiology, Graduate School of Medical Sciences, Kanazawa University, Kanazawa, Ishikawa, Japan; North Carolina State University, Professor, Department of Chemical and Biomolecular Engineering, PO Box 7905, Engineering Building I, UNITED STATES OF AMERICA, Raleigh, NC, 27695

## Abstract

Cell sorting by differential adhesion is one of the basic mechanisms explaining spatial organization of neurons in early stage brain development of fruit flies. The columnar arrangements of neurons determine the large-scale patterns in the fly visual center. Experimental studies indicate that hexagonal configurations regularly appear in the fly compound eye, which is connected to the visual center by photoreceptor axons, while tetragonal configurations can be induced in mutants. We need a mathematical framework to study the mechanisms of such a transition between hexagonal and tetragonal arrangements. Here, we propose a new mathematical model based on macroscopic approximations of agent-based models that produces a similar behavior changing from hexagonal to tetragonal steady configurations when medium-range repulsion and longer-range attraction between individuals are incorporated in previous successful models for cell sorting based on adhesion and volume constraints. We analyze the angular configurations of these patterns based on angle summary statistics and compare between experimental data and parameter fitted ARA (Adhesion-Repulsion-Attraction) models showing that intermediate patterns between hexagonal and tetragonal configuration are common in experimental data as well as in our ARA mathematical model. Our studies indicate an overall qualitative agreement of ARA models in tile patterning and pave the way for their quantitative studies. Our study opens up a new avenue to explore tile pattern transitions, found not only in the column arrangement in the brain, but also in the other related biological processes.

## Author summary

Biological tile patterns often exhibit hexagonal patterns, such as those seen in insect compound eyes, columnar structures in the brain, auditory epithelial cells, and lobules in the liver, which are thought to be due to physical constraints. Indeed, hexagonal tile patterns minimize the potential energy based on a standard physical potential of particles. In this study, we focus on the arrangement of the columnar structure in the fly brain. Our quantitative method to analyze the symmetric distribution patterns of particles shows that their distribution patterns are not exactly hexagonal or tetragonal. We therefore propose a new mathematical model, the ARA model, which takes into account short-range adhesion, medium-range repulsion and long-range attraction and reproduces hexagonal, tetragonal and intermediate patterns. Finally, the ARA model could reproduce the changes in column arrangement upon decreasing and increasing the expression level of N-cadherin. The current study provides a mathematical basis for studying a wide range of tile patterns found in biological and non-biological systems.

## Introduction

Organisms exhibit a variety of tile patterns, such as those seen in insect compound eyes, columnar structures in the brain, auditory epithelial cells, and lobules in the liver. Various cell adhesion molecules regulate cell clustering and cell separation through homophilic and/or heterophilic cell adhesion in the fly compound eye (e.g. Cadherins and Irre Cell Recognition Modules) and mouse auditory epithelium (e.g. Nectins) [2, 3, 21, 54, 55].

These biological tile patterns often exhibit hexagonal patterns, which is often thought to be based on physical restrictions such as short circumference and high space-filling, such as insect compound eyes, liver lobules, and scales in armadillos. However, aquatic arthropods such as shrimp exhibit tetragonal patterns in the compound eye [17]. It is also known that the compound eye of the fruit fly, *Drosophila melanogaster*, normally exhibits a hexagonal pattern, but in some mutant backgrounds it changes to a tetragonal pattern [22]. Thus, organisms can produce either hexagonal or tetragonal tile patterns. A recent study focusing on the *Drosophila* compound eye showed that the combination of tissue-wide tension and cellular growth force, in addition to cell adhesion and physical constraints, plays an important role in the hexagonal to tetragonal transition, suggesting that multiple sources of force are necessary to account for the formation of biological tile patterns [22, 54].

Columnar structures in the brain, which are formed by the cylindrical accumulation of multiple neurons, are the functional units of the brain, and their arrangement patterns are thought to play an important role in brain function. It is known that columnar structures in the fly visual center and microcolumns in the mouse cerebral cortex show a hexagonal arrangement, too [37, 38, 57]. However, the mechanisms controlling the tiling patterns of these columnar structures are unknown. Although hexagonal tile patterns tend to be preferred in the presence of physical constraints, it is also possible that the arrangement of the columns is not simply based on physical stability. If so, it is conceivable that the column arrangement may exhibit not only hexagonal but also tetragonal patterns.

**Fig 1:**
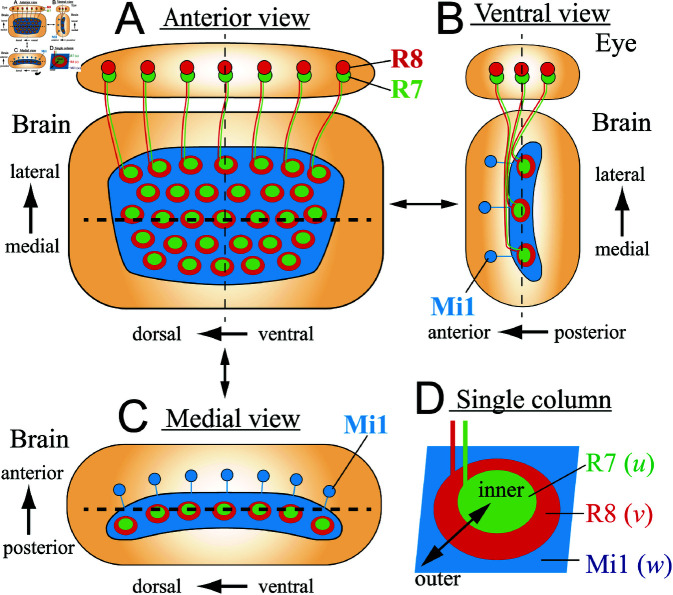
Schematics of the columns in the fly brain. R7 (green) and R8 (red) in the compound eye and Mi1 (blue) in the brain send their axons to the medulla columns. (A) Anterior view shows the two-dimensional column arrangement presented in this paper. The thin and thick broken lines indicate the sections shown in panels B and C, respectively. Mi1 cell bodies are out of focus. (B) Ventral view shows a section indicated by the thin dotted line in Fig A. (C) Medial view shows a section indicated by the thick dotted line in panel A. R7 and R8 cell bodies are out of focus. (D) magnified drawing of a single column.

Compared to the columns in the mammalian brain, which are composed of numerous neurons, fruit fly columns are structurally simpler, consisting of approximately 100 neurons, which receive visual input from the photoreceptor neurons in the compound eye. During early development (larval to early pupal stages), three neurons called R7, R8, and Mi1 play a central role in establishing the basic structure of the column: axons of R7 project to the center of the column and form a dot-like area; axons of R8 surround R7 and form a horseshoe-like circular area; axons of Mi1 surround R8 and occupy a grid-like region as shown in Fig 1.

We found in [57] that the expression level of N-cadherin (Ncad), an evolutionarily conserved cell adhesion molecule, was strong in R7, intermediate in R8, and weakest in Mi1. The cells with stronger adhesion would be located on the inner side and the cells with weaker adhesion would be located on the outer side of the column. In fact, we have shown through a combination of experiments and a mathematical model that the cells are positioned according to the differences in cell adhesion, with R7 on the inner side, R8 just outside of it, and Mi1 at the outermost side [12, 57].

Cell sorting mechanisms have been considered in mathematical biology since the formulation of the differential adhesion hypothesis (DAH) by Malcolm Steinberg [45–48] more than 50 years ago. Differential adhesion between different cell populations [26, 56] is now understood as a fundamental mechanism for cellular patterns, confirmed by experiments [14, 18, 19, 23, 24, 31, 58] and by mathematical models that are able to identify suitable parameters [12, 15, 16, 28, 44]. Mathematical Population Models (MPM) of cell sorting by differential adhesion are derived from Agent Based Models (ABM) by a coarse graining procedure usually referred as the mean-field approximation [9, 11, 12, 36]. MPMs include a nonlocal term incorporating contact-mediated adhesion between cells. Long filopodia or protrusions are observed in experiments that produce contact forces between neighboring cells, see the discussion and derivation from Agent Based Models (ABM) in related contexts of zebra fish pattern formation and tissue morphogenesis [10, 36, 59]. These nonlocal population models can lead to aggregation-diffusion population models if volume effects are taken into account by introducing a strong repulsion at the origin. This approach leads to nonlinear diffusion terms [12, 42] in the population model instead of linear ones early used for this purpose [1]. Nonlinear diffusive terms allow for sharp interfaces between cell populations, being more natural than linear diffusion if population pressure is more important than Brownian motion fluctuations of the cells. In summary, previous works [1, 12, 42] introduced volume constraints by nonlinear diffusion and long range attraction by nonlocal terms with possible proliferation modelling tissue growth introduced by Fisher-KPP terms. Numerical experiments based on this MPM reported in [57] show that differential adhesion is sufficient for concentric organization of the individual columns in the fly visual center.

However, our careful numerical exploration of MPMs for three populations densities (R7, R8 and Mi1) incorporating only differential adhesion via a long-range attractive potential does not lead to a transition between hexagonal to tetragonal tiling patterns. Tetragonal configurations are proven to be the minimizers of the adhesion energy for repulsive-attractive interaction potentials [53], and numerically confirmed in [33]. From a biological perspective, it is known that ligands can lead to attractive or repulsive interaction at medium- or long-range distances depending on the biochemical pathways involved [49, 52, 62].

In this study, we extend the old model of cell sorting based on differential adhesion [45] by incorporating a medium-range repulsion in between short-range adhesion and long-range attraction (ARA model) for the interactions between cells of the most cohesive population, the one with the strongest long-range attraction force – R7 in the case of fly visual center – see Fig 3. Symmetric differential adhesion for the other inter- and intra-cellular interactions is kept – R8 and Mi1 in the case of fly visual center. Using the new ARA model, we have succeeded in reproducing hexagonal, tetragonal, and intermediate patterns interpolating between the two, matching the tailored-designed experimental results. Transitions in the tiling mechanism from tetragonal to hexagonal patterns is explained by varying the strength of the repulsive medium-range force and the length of the terrace between medium-range repulsion and long range attraction for the R7 population confirming the intuition derived from the theoretical results in [53]. We analyze this behavior using angle summary statistics to discern among hexagonal and tetragonal like patterns. Similar strategies based on summary statistics have been used in pattern classification for different purposes in mathematical biology [5, 6, 40, 41].

**Fig 2:**
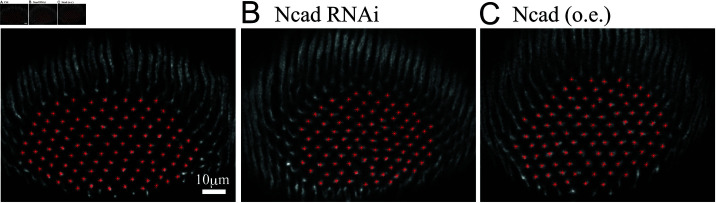
The columnar patterns in the pupal brain. The columnar patterns from the (A) control (*R7-Gal4 UAS-myrGFP*), (B) Ncad RNAi (*R7-Gal4 UAS-myrGFP / UAS-Ncad RNAi*) and (C) Ncad over-expression (o.e.) experiments (*R7-Gal4 UAS-myrGFP / UAS-Ncad*) at 20 hours APF. Anterior views. The columnar positions extracted from them are marked with red asterisks.

## Materials and Methods

### Experimental Data

The core columnar neurons, R7, R8, and Mi1, form concentric domains that establish the basic columnar structure in the larval brain, which grow into a mature three-dimensional column structure during the pupal stage [57]. In the early larval stage, axons of R8, R7, and Mi1 sequentially project to the column, and in the late larval stage, they form a concentric column structure. In this study, the simulation starts when all axons of three types of core columnar neurons project to the column in the late larval stage. In the larval stage, the number of columns is still small and the quantitative results of the column arrangement pattern are not stable. So, we focus on the column arrangement pattern in the brain during the early pupal stage (20 hours after puparium formation: APF). In addition to R7, R8, and Mi1, many other types of neurons project to the columns, but for simplicity, only three types of columnar neurons were considered in this study.

Always centrally located in the column, R7 specifically expresses the cell adhesion molecule Fasciclin II (Fas2) [35, 60]. At 20 hours APF, brains were stained with mouse anti-Fas2 and Cy3-conjugated anti-mouse antibodies, mounted so that the column surface was placed horizontally to the cover glass [20]. The tips of the R7 axons were imaged using a confocal laser microscopy seeing from the anterior side of the brain (Fig 1). After selecting data in which most of the columns were located in the same plane, individual R7 terminals were selected using the *Wand* function of ImageJ. Since the brain is slightly curved, the R7 terminals that are out of focus located at the edge region were excluded from the analysis. The coordinates of their centers of mass were quantified using the *Measure* function, and these were used as the coordinates of the columns.

In addition to wild-type flies, we used a fly strain that expresses a transcription factor Gal4 in R7-specific manner (*R7-Gal4*) and a membrane-localized GFP under the control of Gal4 (*UAS-myrGFP*), which visualizes axons [4, 57]. The *R7-Gal4 UAS-myrGFP* fly was crossed with either wild-type (Ctrl), *UAS-Ncad RNAi*, which knocks down Ncad under Gal4 control (Ncad RNAi), and *UAS-Ncad*, which overexpresses Ncad (Ncad (o.e.)) [57]. Thus, we artificially reduced and enhanced the function of Ncad in R7-specific manner and obtained column coordinates as above.

The distribution of the columns as visualized by Fas2 staining appeared to be hexagonal in control brains, see Fig 2A, whereas the pattern showed some irregularity when Ncad was either knocked-down or over-expressed in R7, see Fig 2B and 2C. To quantify the spatial pattern distribution of the columns, we formulate a quantitative method to analyze the symmetries of image data as explained below in the next sections.

### Model Background: Cell-Cell adhesion & Symmetry Patterns for Multiple Populations

As in [12], we introduce the population dynamics model for a single species in two spatial dimension [12] that reads as:

$$\frac{\partial u}{\partial t}=\nabla \cdot \left(u\nabla u\right)-\nabla \cdot \left(u(1-u)\text{K}(u)\right),$$ (1)

$$\text{K}(u)(\text{x})=\int_{0}^{1}\;\;\;\int_{S^{1}}[u(\text{x}+r\eta )\omega (r)]r\eta \;d\eta dr,$$

where u(x,t) denotes the population density of cells at spatial position $\text{x}\in {\mathbb{R}}^{2}$ and time *t*, ${\text{S}}^{1}$ is the unit circle and ω is a function that controls adhesion and repulsion between cells according to the distance from x. The first term on the right-hand side of (1) denotes the basic behavior of cells, in which cells move from areas with high cell densities to areas with low cell densities due to population pressure. The integral term K(u) of the second term represents that each cell perceives the situation within its own sensing radius *R* and moves in the desired direction accordingly. The sensing radius *R* is normalized to 1. The preference according to distance from itself is expressed by the interaction kernel ω. The term 1–*u* represents the effect of density saturation. We showed that the nonlinear diffusion, first term in (1), is crucial to have sharp interfaces instead of diffuse interfaces [1]. Moreover, this model is able to capture a good deal of mixing/sharp boundary behavior in tissue growth experiments [42]. Furthermore, Carrillo *et al*. [12] observed pattern formation by short-range adhesion and middle-range repulsion using (1). In particular, hexagonal spot patterns of cell populations have been ubiquitously obtained.

**Fig 3:**
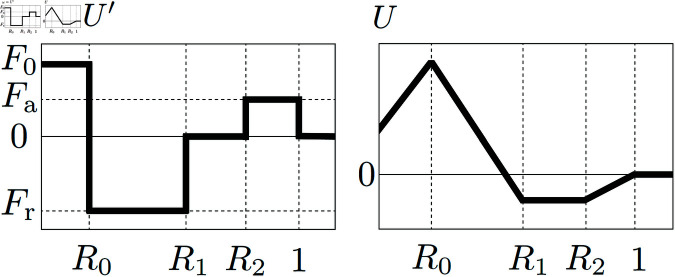
The shapes of the interaction kernel ω(r) and the corresponding potential U(r). The parameters involved correspond to forces stregths: *F*_0_ magnitude of adhesion, *F*_*a*_ magnitude of attraction, and *F*_*r*_ magnitude of repulsion; while the radii *R*_0_, *R*_1_, and *R*_2_ determine the different regions: short-range adhesion ($0\leq r<R_{0}$), medium-range repulsion ($R_{0}\leq r<R_{1}$), and long-range attraction ($R_{2}\leq r<1$).

Theil [53] treated rigorously the geometric properties of the ground-state configuration of many-particle systems in two spatial dimensions under suitable assumptions on the interaction potential. He pointed out that the triangular lattice does not always have the lowest energy even for natural interaction potentials, and provided an interaction potential such that the energy per particle of the triangular lattice is higher than that of the square lattice. Based on his considerations, we use a simple potential. While there are many reasonable choices for potentials to investigate biological phenomena of interest, we introduce now the novel ARA model by defining the kernel ω(r). In order to simplify the parameter estimation, in this paper, we deal with a piecewise linear potential *U* and hence the following piecewise constant kernel $\omega =U^{\prime }$ as in Fig 3 defined by:

$$\omega (r)=\left\{ \begin{array}{cc} F_{0} & \text{if }0\leq r<R_{0},\\ F_{r} & \text{if }R_{0}\leq r<R_{1},\\ 0 & \text{if }R_{1}\leq r<R_{2},\\ F_{a} & \text{if }R_{2}\leq r<1,\\ 0 & \text{if }1\leq r. \end{array}\right.$$ (2)

**Fig 4:**
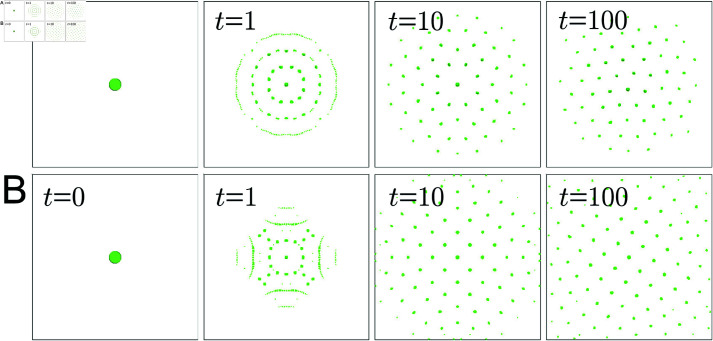
Numerical results for the ARA model (1)–(2). The parameters are (A) *R*_0_ = 0.14, *R*_1_ = 0.55, *R*_2_ = 0.58, *F*_0_ = 5000, $F_{r}=-5000$, $F_{a}=500$. (B) *R*_0_ = 0.14, *R*_1_ = 0.55, *R*_2_ = 0.88, *F*_0_ = 5000, $F_{r}=-4000$, $F_{a}=500$.

This kernel incorporates short-range adhesion ($0\leq r<R_{0}$), medium-range repulsion ($R_{0}\leq r<R_{1}$), and long-range attraction ($R_{2}\leq r<1$). The parameters *F*_0_>0, $F_{r}<0$ and $F_{a}>0$ control the strengths of cell-cell adhesion, that of repulsion and that of attraction, respectively. Motivated by Theil’s considerations, the terrace U(r)=const., i.e., $\omega (r)=0\;(R_{1}\leq r<R_{2})$ is introduced. Due to the balance of adhesion, repulsion, attraction and terrace, cells form small clusters, and these clusters are expected to arrange in a triangular or square lattice. The behavior induced by the ARA model is verified through numerical calculations. To this end, an initial condition is set where cells are gathered at one location, as shown in Fig 4. This initial condition is artificially chosen and not based on biological data. By handling this initial condition, we can observe the process in which cells spontaneously form small clusters, and these clusters naturally align in regular patterns.

Throughout this work, all numerical simulations are carried out in a 2D domain (−2.8,2.8)^2^ with periodic boundary conditions. The problems are discretized by the standard explicit upwind finite volume method [8], and the nonlocal terms are calculated by numerical integrations explained by Murakawa and Togashi [42]. The initial value is *u*_0_ = 0.1 inside a circle with a center at the origin and a radius of 0.2, and *u*_0_ = 0 otherwise. Grids where u≥0.01 are drawn in green and grids where *u* < 0.01 are painted in white. Therefore, the green areas represent clusters of cells. Fig 4 shows the numerical results with two sets of parameters.

We observe that cells gathered at the origin are scattered by medium-range repulsion, but form small clusters by short-range adhesion. The small clusters are arranged regularly due to the balance between medium-range repulsion and long-range attraction. In Fig 4A, cell clusters are arranged in a triangular lattice, and in Fig 4B, cell clusters are arranged in a square lattice. The main difference between the two is the size of the terrace, *i.e. R*_2_.

Although in our experimental setting in the previous section only R7 was visualized in biological experiments, individual columns also contain R8 and Mi1. Thus, we extend the single species model to the three-species model representing the densities of R7, R8, and Mi1, as described in [12, 57]. Our three-species mathematical model representing the cell densities *u*,*v*,*w* of R7, R8, and Mi1, respectively, is formulated as follows:

$$\left\{ \begin{array}{c} \frac{\partial u}{\partial t}=\nabla \cdot \left(u\nabla \sigma \right)-\nabla \cdot \left(u(1-\sigma ){\text{K}}_{\;1}(u,v,w)\right),\\ \frac{\partial v}{\partial t}=\nabla \cdot \left(v\nabla \sigma \right)-\nabla \cdot \left(v(1-\sigma ){\text{K}}_{\;2}(u,v,w)\right),\\ \frac{\partial w}{\partial t}=\nabla \cdot \left(w\nabla \sigma \right)-\nabla \cdot \left(w(1-\sigma ){\text{K}}_{\;3}(u,v,w)\right). \end{array}\right.$$ (3)

Here, the total cell density of neurons is denoted by σ=u+v+w. This Partial Differential Equations (PDEs) model includes volume size constraints in the form of total population pressure, nonlocal interactions incorporating attraction and repulsion, and density saturation effects. We refer to [12] for the full mathematical description. The nonlocal interactions ${\text{K}}_{\;i}$ (i=1,2,3) are fully specified later.

### Symmetry Indices & Data analysis

**Fig 5:**
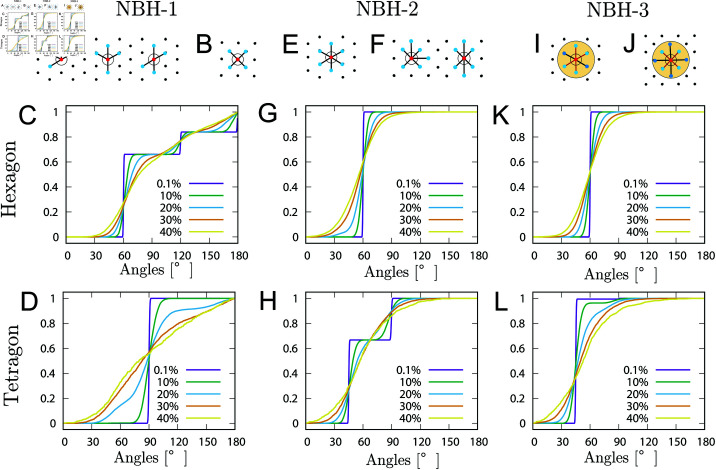
Nearest neighborhood types and cumulative angle distributions for perturbed patterns. (A), (B), (E), (F), (I) and (J): sketch of three typical types of nearest neighborhoods. (C), (D), (G), (H), (K) and (L): cumulative angle distribution functions using these three types of neighborhoods for hexagonal and tetragonal patterns with perturbations ranging from 0.1% to 40%.

In order to analyze the symmetries on experimental and/or synthetic data, we introduce three symmetry indices. We first take a particle approximation both of the data and the simulations obtained with our continuum model. Similarly to the experimental image analysis, the center of the local density peaks of R7 neurons in our simulations using the PDE model (3) is also directly obtained by its center of mass. Once we have this particle data approximation, we calculate the angle formed by each particle with its neighboring particles, and analyse the angle statistical distribution. There are various ways to determine angles of a home particle with neighboring particles, but we deal with the following three procedures:

- **NBH-1:** Consider the angle formed by the home particle and its 4 nearest neighbors.
  A slight perturbation of a perfect hexagonal pattern should produce angles close to those in Fig 5A, and the angle statistical distribution should concentrate at 60, 120, and 180 degrees as shown in Fig 5C. On the other hand, a slight perturbation of a perfect tetragonal pattern should only lead to angles like those in Fig 5B, and the angle statistical distribution should be concentrated at 90 degrees as shown in Fig 5D. The graphs in Fig 5C and 5D represent the cumulative distribution functions of angles when perturbations are applied to 4096 particles arranged in a perfect hexagonal or square configuration pattern. These perturbations are realized by adding random displacements to the coordinates of individual lattice points. The maximum displacement is given in advance as a percentage of the interparticle distance.
- **NBH-2:** Consider the angle formed by the home particle and its 6 nearest neighbors.
  A slight perturbation of a perfect hexagonal pattern should only present angles close to those in Fig 5E. Therefore, the angles statistical distribution should be concentrated at 60 degrees as shown in Fig 5G. On the other hand, a slight perturbation of a perfect tetragonal pattern should only present angles close to those in Fig 5F, and the angle statistical distribution should concentrate around 45 and 90 degrees as shown in Fig 5H.
- **NBH-3:** Consider the angle formed by the home particle and the particles located in a circular region centered at the home particle.
  The circular region is specified by taking the average $d^{ave}$ of the distances between the home particle and its 4 nearest neighbors. Consider all particles within a distance of $d^{ave}$ × $\sqrt{2}\alpha_{1}$ from the home particle. Then, calculate the angle formed between the home particle and each of these particles. In this paper, we set $\alpha_{1}=1.1$. Fig 5I and 5J show examples for the hexagonal and tetragonal patterns, respectively. The lengths of the red bonds are the distance between the home particle and its four nearest neighbors, and the range is drawn in light green.
  For a slight perturbation of a perfect hexagonal pattern, the number of particles within the circular neighborhood should be 6, and the angle statistical distribution must concentrate at 60 degrees as shown in Fig 5K. On the other hand, for a slight perturbation of a perfect tetragonal pattern, the number of particles within the circular region should be 8, and the angle statistical distribution should be concentrated at 45 degrees as shown in Fig 5L.

**Fig 6:**
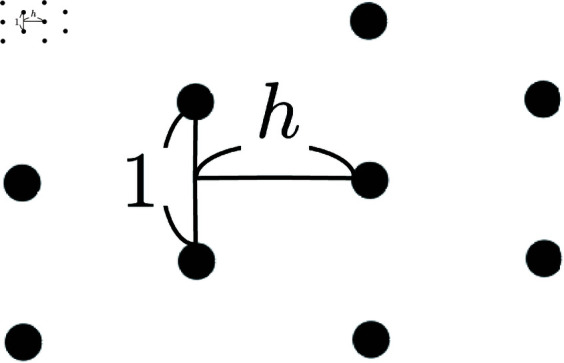
Scheme of the regular lattice and the parameter h.

The arrangement of the columns is analyzed by comparing the angle distribution function of experimental/numerical data with those of perturbations applied to regular lattices. The regular lattice discussed here is the one shown in Fig 6, and is a one-parameter family parameterized by *h*. It is the hexagonal lattice when $h=\sqrt{3}/2$, the square lattice when *h* = 1/2, and a centered rectangular lattice otherwise.

Let $F_{k}^{data}(\theta )$ (k=1,2,3) be the cumulative statistical distribution function of angles using NBH-*k* obtained from the position of the column based on experimental/numerical data, and let $F_{k}(\theta ;h,p)$ be the cumulative distribution function of angles of the perturbed regular lattice with a parameter *h* and a disturbance p%. We calculate the ${\text{L}}^{2}$ error between $F_{k}^{data}$ and $F_{k}(\cdot \;;h,p)$, and define the numerical pair $\left(h_{k}^{*},p_{k}^{*}\right)$ that minimizes this difference, namely,

$$\begin{array}{ccc} E_{k}(h,p) & = & \|F_{k}^{data}-F_{k}(\cdot \;;h,p){\|}_{L^{2}(0,\pi )},\\ \left(h_{k}^{*},p_{k}^{*}\right) & = & {\mathrm{\backslash argmin}}_{0.5\leq h\leq 1,\;0\leq p\leq p_{max}}E_{k}(h,p). \end{array}$$ (4)

Here, $p_{max}$ is a given maximum value of disturbance. Let the value of $h_{k}^{*}$ be denoted by Index-*k*.

Notice that since the number of particles is finite, a careful attention must be paid to the edges of the particle clusters. Calculate the distance *d*_*i*_ from each particle named *i* to its nearest neighboring particle, and let the median of *d*_*i*_ be denoted as $d^{med}$. If there are 6 or more particles within a radius of $d^{med}\sqrt{2}$ × $\alpha_{2}$ from a particle, that particle can be considered as a home particle and angles around it are calculated. Otherwise, it is regarded as an edge particle and therefore angles around it are not calculated. To identify diagonally neighboring particles within a tetragonal lattice, while excluding those separated by a distance around $2d^{med}$ (i.e., particles two positions away), we account for approximately 20% perturbations, setting $\alpha_{2}=1.2$ as the threshold for diagonal neighbor consideration. This threshold ensures the reliable capture of relevant neighbors while excluding more distant particles.

Combining multiple spatial statistics is a successful method to enhance pattern identification in image analysis [5–7, 36]. Regarding the three indices (Index-1, Index-2, Index-3), none of them is fully conclusive for detecting the correct angle for general images. If similar conclusions can be obtained using different indices, it supports the final assertion. We analyse the performance of each index. While the three indices are technically equivalent, Index-3 has the advantage that it does not consider a specific number of neighbors in advance. We will use Index-3 to perform a sensitivity analysis in Figs 10–12.

**Fig 7:**
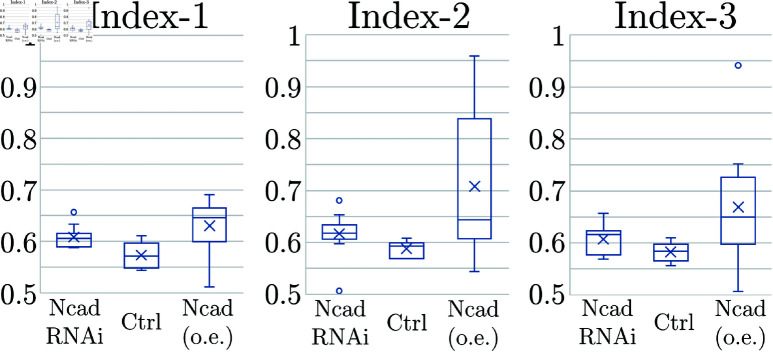
Comparison of Indices-1,2,3 from experimental data sets. Boxplots of Indices-1,2,3 in 5 Ctrl, 11 NcadRNAi and 8 Ncad (o.e) samples. The variability of the data is represented using standard boxplots, with the mean of each dataset shown as a cross. The whiskers extend to 1.5 times the interquartile range, and any data points outside this range, marked as circles, are considered outliers. Note that the indices are $\sqrt{3}/2$ for hexagonal patterns and 1/2 for tetragonal patterns.

**Fig 8:**
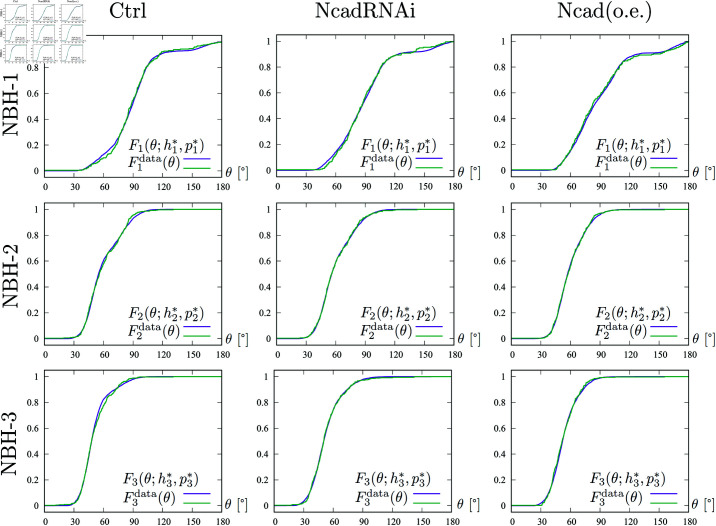
Cumulative angle distributions of columnar patterns in different experimental conditions. The cumulative distribution functions of the angles of the columnar patterns from the control, NcadRNAi and Ncad (o.e) experiments in Fig 2 corresponding to NBH-1–3, respectively. The two lines represent $F_{k}^{data}(\theta )$ obtained from experimental data (green) and $F_{k}(\theta ;h_{1}^{*},p_{1}^{*})$ a perturbed regular lattice fitted to the experimental data (magenta).

**Fig 9:**
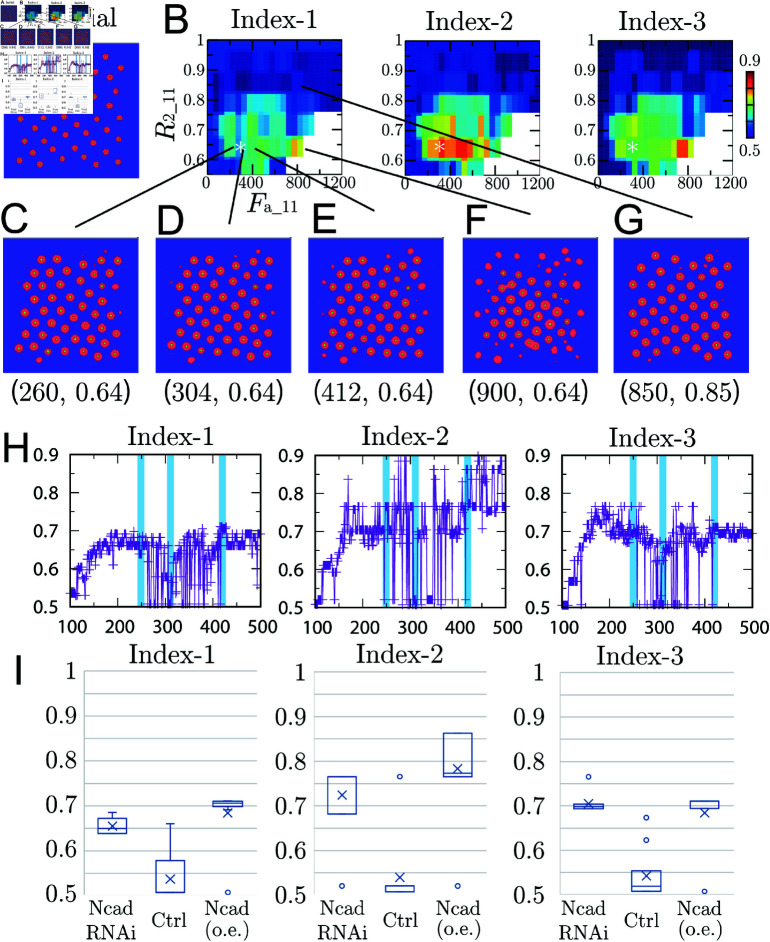
Numerical results for the ARA model using different parameters $F_{a\_11}$ and $R_{2\_11}$. (A) Initial state based on experimental data. (B) Indices using different parameters $F_{a\_11}$ and $R_{2\_11}$ at which numerical solutions were reached numerical steady states. The asterisk * indicates the values fixed in Figs 11 and 12. (C)–(G) Several selected numerical steady states with parameters ($F_{a\_11}$, $R_{2\_11}$) using the color code in Fig 1. (H) Indices using different parameters $F_{a\_11}$ with fixed $R_{2\_11}=0.64$ at which numerical solutions were reached numerical steady states. (I) Boxplots of Indices-1,2,3 from $F_{a\_11}=245\text{--}254$ (labeled NcadRNAi), $F_{a\_11}=305\text{--}314$ (labeled Ctrl) and $F_{a\_11}=415\text{--}424$ (labeled Ncad (o.e.)) numerical data sets. Each dataset contains 10 data points. These parameter ranges are highlighted as light blue bands in panel H. Each average value is indicated by a cross, and in panels C–E, results using data close to each average value are plotted.

**Fig 10:**
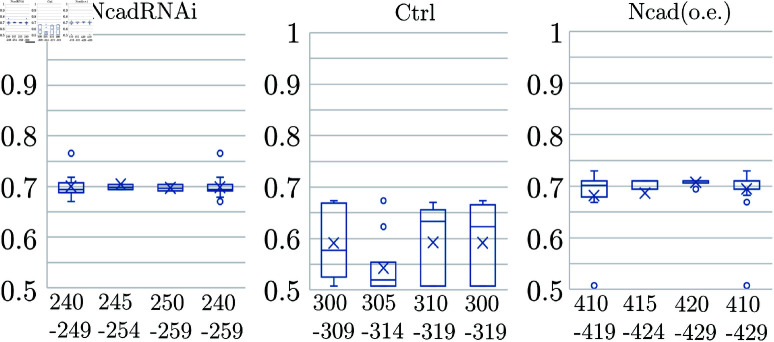
Boxplots of Index-3 values showing the effects of variations in the range and position of $F_{a\_11}$. The parameter range is varied for NcadRNAi, Ctrl, and Ncad (o.e.). The specific parameter ranges are indicated below the figure.

**Fig 11:**
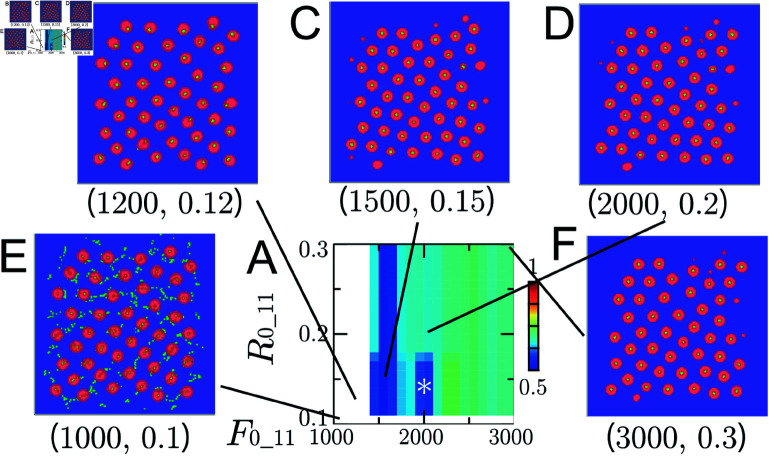
Numerical results for the ARA model using different parameters $F_{0\_11}$ and $R_{0\_11}$. (A) A plot of Index-3 with different parameters, $F_{0\_11}$ and $R_{0\_11}$, at which numerical solutions were reached numerical steady states. The asterisk * indicates the values fixed in Numerical Simulations section. (B)–(F) Several selected numerical steady states with parameters ($F_{0\_11}$, $R_{0\_11}$) using the color code in Fig 1.

**Fig 12:**
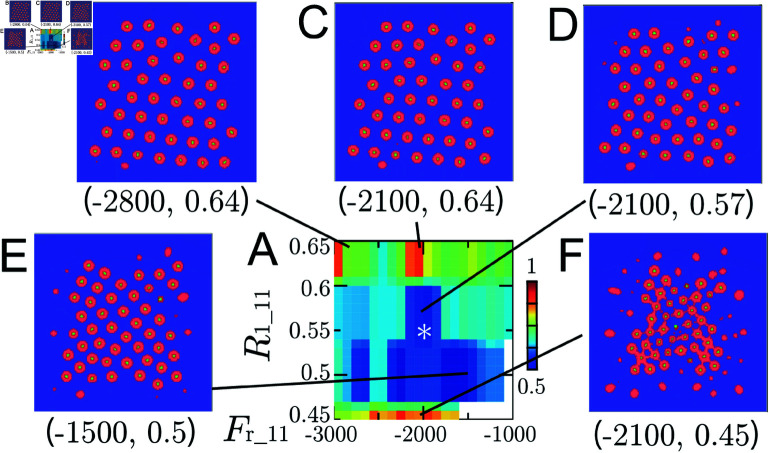
Numerical results for the ARA model using different parameters $F_{r\_11}$ and $R_{1\_11}$. (A) A plot of Index-3 with different parameters, $F_{r\_11}$ and $R_{1\_11}$, at which numerical solutions were reached numerical steady states. The asterisk * indicates the values fixed in Numerical Simulations section. (B)–(F) Several selected numerical steady states with parameters ($F_{r\_11}$, $R_{1\_11}$) using the color code in Fig 1.

## Results

### Analysis of Symmetries on Experimental Data

We analyze five control (Ctrl), eleven Ncad knockdown (NcadRNAi) and eight Ncad over-expression (Ncad (o.e)) data sets using Indices-1,2,3. The results are summarized in Fig 7.

In order to demonstrate the reliability of the indices, we pick up data with index 3 close to the average value one by one from the Ctrl, NcadRNAi, and Ncad (o.e) datasets, and plot them in Fig 2. We plot the cumulative distribution functions of the angles corresponding to NBH-1–3 in Fig 8. The two lines represent the cumulative distribution functions of the angles $F_{k}^{data}(\theta )$ obtained from experimental data and $F_{k}(\theta ;h_{1}^{*},p_{1}^{*})$ of a perturbed regular lattice as in Fig 6 fitted to the experimental data. The obtained symmetry index Index-$k=h_{k}^{*}$, the corresponding disturbance $p_{k}^{*}$ and error $E(h_{k}^{*},p_{k}^{*})$ obtained by the optimization procedure described in (4) with $p_{max}=40$ are summarized in Table 1. Fig 8 and Table 1 demonstrate that the ${\text{L}}^{2}$-error between the cumulative distribution functions of the experimental data and the ones obtained by the optimization procedure in (4) are less than 10^−2^, see third component of each element in Table 1. The error of this fitting algorithm corroborates the ability of our symmetry indices to measure how close to tetragonal or hexagonal symmetry the experimental data is.

**Table 1:** The parameters and errors $\left(h_{k}^{*},p_{k}^{*},E(h_{k}^{*},p_{k}^{*})\right)$ used in Fig 8.

	Ctrl	NcadRNAi	Ncad (o.e)
Index-1	(0.5735, 0.1900, 0.006781)	(0.6157, 0.1872, 0.009073)	(0.6480, 0.1764, 0.005705)
Index-2	(0.5962, 0.1864, 0.007195)	(0.6135, 0.2040, 0.004349)	(0.6502, 0.1908, 0.004191)
Index-3	(0.5765, 0.1788, 0.006575)	(0.6162, 0.2152, 0.005594)	(0.6507, 0.1764, 0.006320)

In contrast to our initial naive impression that the distribution of the columns is hexagonal in control brains, see Fig 2, the results of our symmetry analyses shown in Fig 7 indicate that the pattern is always at an intermediate state between hexagonal and tetragonal, which was also the case in a common wild-type strain, Canson-S. Furthermore, the control pattern is rather tetragonal (lower indices) while the patterns found in NcadRNAi and Ncad (o.e.) conditions are rather hexagonal (higher indices). We next asked if a similar transition of distribution patterns can be reproduced in numerical simulations.

### Numerical Simulations

In order to investigate how the strength of the attraction/repulsion affects the column arrangement, we carry out numerical experiments based on the ARA mathematical model (3) where the nonlocal interactions ${\text{K}}_{\;i}$ (i=1,2,3) are given by


$${\text{K}}_{\;i}(u,v,w)(\text{x})$$

$$=\int_{0}^{1}\;\;\;\int_{S^{1}}\;[u(\text{x}+r\eta )\omega_{i1}(r)+v(\text{x}+r\eta )\omega_{i2}(r)+w(\text{x}+r\eta )\omega_{i3}(r)]r\eta \;d\eta dr.$$

Since only the adhesion of R7 was artificially manipulated in the experiment, the ARA kernel is employed only for the interaction between R7 cells, while the cell adhesion kernels are used for other interactions, namely,

$$\omega_{11}(r)=\left\{ \begin{array}{cc} F_{0\_11} & \text{if }0\leq r<R_{0\_11},\\ F_{r\_11} & \text{if }R_{0\_11}\leq r<R_{1\_11},\\ 0 & \text{if }R_{1\_11}\leq r<R_{2\_11},\\ F_{a\_11} & \text{if }R_{2\_11}\leq r<1,\\ 0 & \text{if }1\leq r, \end{array}\right.$$ (5)

$$\omega_{ij}(r)=\left\{ \begin{array}{cc} F_{0\_ij} & \text{if }0\leq r<R_{0\_ij},\\ 0 & \text{if }R_{0\_ij}\leq r \end{array}\right.\;(i,j)\neq (1,1).$$ (6)

Numerical simulation using the larval column distribution pattern as the initial condition is difficult because the entire tissue grows during the larval stage and the number of columns itself increases. Therefore, we used the column coordinates of control flies at 20 hours APF as the initial conditions. More precisely, the initial values are constructed as follows. Datum on the column center positions is obtained from a single image at 20 hours APF. For each column center, the distance to the nearest column center is measured, and the average of these distances is calculated. The column centers are then rescaled and repositioned so that this average distance becomes 0.61. This distance is taken such that the columns interact with each other via a kernel $\omega_{ij}$ in (5) for various parameters. The initial values are set as follows: within a distance of 0.04 from each column center, *u* = 0.2; outside this range, *u* = 0; within a distance between 0.04 and 0.12, v=0.1; otherwise, v=0; at distances of 0.12 and above, *w* = 0.04; and below 0.12, *w* = 0. The configuration is shown in Fig 9A.

There are many parameters in the kernel (5), but our purpose is to investigate the effect of attraction on column arrangement. Our previous study revealed long cellular protrusions (filopodia) emanating from the columnar neurons such as R7. Ncad located in the filopodia may mediate a long range attraction [57]. From the data in Experimental Data section, we suggest that the attraction between R7 cells plays an important role in the formation of cell arrangement patterns. Note that forces between two different cell populations with indices *i* and *j* should follow the action-reaction law according to Newton’s third law of motion. Namely, $F_{0\_ij}=F_{0\_ji}$ for all i,j∈{1,2,3}. Similarly, the sensing radius of the force should also be the same. Namely, $R_{0\_ij}=R_{0\_ji}$. Adhesion between three cell types $\left(F_{0\_ij}\right)$ follow the following order according to our previous study [57], since the cells with stronger adhesion are located on the inner side of the column: $F_{0\_11}>F_{0\_12}=F_{0\_21}>F_{0\_22}>F_{0\_23}=F_{0\_32}>F_{0\_33}$; allowing us to preserve the column structure. As seen in Model Background section, the following parameters are fixed for numerical exploration to ensure that R7 maintains multiple small clusters: $F_{0\_11}=2000$, $F_{r\_11}=-2000$, $R_{0\_11}=0.14$, $R_{1\_11}=0.55$. Finally, we set $F_{0\_22}=25$, $F_{0\_12}=F_{0\_21}=50$, $F_{0\_33}=10$, $F_{0\_13}=F_{0\_31}=20$, $F_{0\_23}=F_{0\_32}=15$. Since R8 tends to wrap around R7, then $R_{0\_12}=R_{0\_21}$ is made larger than the others. Therefore, we fix $R_{0\_12}=R_{0\_21}=0.3$, otherwise $R_{0\_ij}=R_{0\_ji}=0.14$.

We next vary $F_{a\_11}$ and $R_{2\_11}$, the parameters that respectively determines the magnitude and range of attractive force between R7, while keeping other parameters fixed. The rationale is the following: Ncad is the only factor we know that can influence the column arrangement through long range attraction via the filopodia. Since we can artificially manipulate the expression level of Ncad (related to $F_{a\_11}$) and the actual range of Ncad action is uncertain (related to $R_{2\_11}$), we only changed $F_{a\_11}$ and $R_{2\_11}$. A table with all parameters can be seen in Table 2. To determine that the numerical solution has approached the steady state, the computation was stopped if the $l^{2}(\Omega )$ error between the numerical solutions at time *t* and at *t*–1 falls below a tolerance 10^−5^.

**Table 2:** Biological interpretation and numerical values of the parameters used in Fig 9.

Parameter	Numerical Value	Biological interpretation
$F_{0\_11}$	2000	Magnitude of adhesion between R7
$F_{r\_11}$	-2000	Magnitude of repulsion between R7
$F_{a\_11}$	variable	Magnitude of attraction between R7
$R_{0\_11}$	0.14	Range of adhesion between R7
$R_{1\_11}$	0.55	Range of repulsion between R7
$R_{2\_11}$	variable	Range of attraction between R7
$F_{0\_22}$	25	Magnitude of adhesion between R8
$F_{0\_12}=F_{0\_21}$	50	Magnitude of adhesion between R7 and R8
$F_{0\_33}$	10	Magnitude of adhesion between Mi1
$F_{0\_13}=F_{0\_31}$	20	Magnitude of adhesion between R7 and Mi1
$F_{0\_23}=F_{0\_32}$	15	Magnitude of adhesion between R8 and Mi1
$R_{0\_12}=R_{0\_21}$	0.3	Range of adhesion between R7 and R8
$R_{0\_ij}=R_{0\_ji}$	0.14	Range of adhesion between the other cell types

Numerical calculations are performed from the experimental column coordinate, that is shown in Fig 9A, for each parameters $F_{a\_11}$ and $R_{2\_11}$. The indices of the columnar coordinates are calculated when sufficient time has passed, and the results are depicted in Fig 9B. The values of the indices are displayed in colors. Here, we set the maximum value of disturbance in (4) as $p_{max}=30$. Additionally, if the columnar pattern is significantly disrupted, it is painted in white. The numerical columnar coordinates for several selected parameters are displayed in Fig 9C–9G. Highly organized tetragonal pattern is observed when $\left(F_{a\_11},R_{2\_11}\right)$ is (850,0.85), see Fig 9G. On the other hand, in the vicinity of $R_{2\_11}=0.64$, differences in the indices can be observed due to variations in $F_{a\_11}$. Even though the indexes are different, their appearance does not change significantly, see Fig 9C–9E. It becomes clear that a quantitative perspective is necessary. When the attraction is too strong, it can be observed that the columnar structure breaks down, see Fig 9F.

Fig 9H shows the details of the change in the indices when $F_{a\_11}$ is changed while $R_{2\_11}=0.64$ is fixed. Although the simulations are performed using a deterministic model under the same initial conditions and the parameters are varied quasi-continuously, the noise is significantly large. This is partially due to the fact that the kernels $\omega_{ij}$ are defined as discontinuous functions, the initial configurations, and the selection of the parameter $\alpha_{2}$, which determines edge particles. Furthermore, when the disturbance $p_{k}^{*}$ is large, as shown in Fig 5, the cumulative distribution function becomes more gradual, making it difficult to determine the pattern, adding further fluctuations. In fact, reducing $p_{max}$ in (4) leads to a decrease in noise.

It is assumed that $F_{a\_11}$ between 305 and 314 corresponds to the control state, and boxplots are made in Fig 9I including cases where the attractions are weak ($F_{a\_11}=245\text{--}254$) and strong ($F_{a\_11}=415\text{--}424$). In Fig 9H, calculations are performed by incrementally changing the value of $F_{a\_11}$ by one. From these data, we found a location where the change in Index-1 with respect to $F_{a\_11}$ was convex, and selected a range of width 10 as corresponding to each of NcadRNAi, Ctrl, and Ncad (o.e.). Although those are based on artificial numerical experiments and the results depend on the initial states, it is observed that the pattern deviates from the tetragonal pattern due to suppression and augmentation of attraction forces similar to the results in Fig 7. Certainly, if we change the range of these parameters $F_{a\_11}$, particularly the center of the blue bands in Fig 7H, different results would be obtained. However, Fig 10 shows that the qualitative results are not sensitive to the variations of the center and the width of the parameter range. Here, the purpose is to demonstrate that increasing or decreasing the attraction force $F_{a\_11}$ may cause shifts in the pattern, similar to the experimental results. It is technically difficult to measure real parameters such as $F_{a\_11}$, and there is no guarantee that the parameters we found above directly correspond to the real phenomenon. Nevertheless, the method we developed opens an avenue to study biological tiling patterns in a quantitative manner.

### Role of Adhesion and Repulsion Parameters

In this section, numerical experiments are conducted by varying $F_{0\_11}$ and $R_{0\_11}$, as well as $F_{r\_11}$ and $R_{1\_11}$. While $F_{a\_11}$ and $R_{2\_11}$ specify the magnitude and range of the attractive force between R7, $F_{0\_11}$ and $R_{0\_11}$ control those of the adhesion force between R7. Similarly, $F_{r\_11}$ and $R_{1\_11}$ determine those of the repulsive force between R7. Here, $F_{a\_11}=300$ and $R_{2\_11}=0.64$ are fixed (Fig 9B), and the initial condition and other fixed parameters are taken from Numerical Simulations section.

The distribution of Index-3 when $F_{0\_11}$ and $R_{0\_11}$ are varied is shown in Fig 11A, and the corresponding numerical columnar coordinates are illustrated in Fig 11B–11F. For small adhesion forces (Fig 11E), R7 cannot form cohesive clusters and scatters. When the adhesion force is slightly increased (Fig 11B), R7 forms clusters, but these clusters shift away from the center of the R8 clusters, failing to maintain the regular columnar structure (see Fig 1D). While the parameters controlling adhesion, $F_{0\_11}$ and $R_{0\_11}$, are essential for maintaining the columnar structure, their overall impact on columnar coordinates appears limited, as seen in Fig 11C, 11D and 11F.

The distribution of Index-3 when $F_{r\_11}$ and $R_{1\_11}$ are varied is depicted in Fig 12A, and the corresponding numerical columnar coordinates are shown in Fig 12B–12F. Here, the role of the terrace, that is the region where $\omega_{11}(r)=0$ for $R_{1\_11}\leq r<R_{2\_11}$, becomes apparent. When $R_{1\_11}$ is close to $R_{2\_11}$ (Fig 12B and 12C), forming a negligible terrace, the columnar coordinate takes a form of a hexagonal structure. With a larger terrace, i.e., when $R_{1\_11}$ is small (Fig 12D and 12E), tetragonal coordinates become dominant. If the repulsive force is insufficient (Fig 12F), columns stick together, and the coordinate fails to maintain proper columnar structures. The parameters controlling repulsion, $F_{r\_11}$ and $R_{1\_11}$, are necessary for maintaining columnar coordinates. Furthermore, terraces of sufficient width enable the emergence of tetragonal, hexagonal, or intermediate structures.

## Discussion

### Hexagon, Tetragon and Something in Between

Biological tile patterns often exhibit hexagonal patterns, which is thought to be due to physical constraints. However, the compound eyes of shrimp change from hexagonal to tetragonal patterns during development [17]. The compound eye of the fruit fly, which normally shows a hexagonal pattern, changes to a tetragonal pattern in mutant backgrounds [22]. Thus, organisms can produce either hexagonal or tetragonal tile patterns. Additionally, a variety of tissues may show a wide variety of tile patterns that are not simply hexagonal or tetragonal. Indeed, the sunflower seed pattern is a complex of diverse tile patterns that range from hexagons and tetragons [61]. Thus, intermediate patterns between hexagonal and tetragonal may also be common, as seen in the columns in the fly brain.

### Quantification of Column Arrangement

In this study, we have developed a quantitative method to analyze the symmetric distribution patterns of cellular units such as columns. Contrary to our intuition that the column pattern is hexagonal, the quantification results of the control patterns showed that they are not exactly hexagonal or tetragonal. The indices indicate that the actual tile pattern is somewhere between hexagonal and tetragonal, and is more similar to tetragonal (Fig 7, Table 1). Ncad knockdown and Ncad overexpression in R7 resulted in deformations of the tile pattern (Fig 2). The quantification of these patterns suggested that the patterns became more similar to hexagonal by decreasing and increasing the expression levels of Ncad (Fig 7). The same method may be used to quantify the distribution pattern of microcolumns in the mouse cerebral cortex [37].

Here, we emphasize that finding alternative indices for angle statistics to improve the data analysis of hexagonal versus tetragonal tile patterns is a timely research topic. Similar strategies for fitting parameters using summary statistics, pattern simplicity scores or persistent-homology approaches have been used to characterize pattern formation in several mathematical biology models, see [5–7, 36, 40, 41] for instance.

### Development of ARA Model

Based on a standard physical potential of particles such the Lennard-Jones potential, hexagonal tile patterns minimize the potential energy. Although hexagonal tile patterns are common in biology, there are many other variations, including tetragonal and something in between hexagonal and tetragonal, as discussed above. Theil proposed that a modification of the physical potential by extending the low-potential area allows the tetragonal configurations [53]. To make such unconventional potentials compatible with biological molecules, we considered the combination of medium-range repellent and long-range attractant, which can modify the extent of the low-potential area. In this study, we developed the ARA model to deal with variation of the tile patterns found in the fly brain. The ARA incorporates medium-range repulsion embedded between short- (contact-mediated) and long-range attraction, and reproduces different tile patterns composed of multiple species (or multiple cell types). Interestingly, a recent study revealed a similar configuration of the potential energy of cell-cell interactions by a statistical inference from cell tracking data [30].

We focused on the function of a cell adhesion molecule, Ncad, which is thought to mediate contact-mediated adhesion, or short-range attraction. However, our previous study revealed long cellular protrusions (filopodia) emanating from the columnar neurons such as R7 [57]. Considering that cell adhesion can be mediated by Ncad located in the filopodia, Ncad-mediated adhesion may occur at a long range and is compatible with the long-range attraction, which is related to the parameter $F_{a\_11}$ in the numerical experiments performed in this study.

### Future Studies

Although we focused on the function of Ncad in this study, the defects caused by decreasing and increasing the expression levels of Ncad were somewhat minor. There must be many additional guidance molecules that regulate medium-range repulsion and long-range attraction. Slit, Semaphorin, Wnt, and Netrin are typical families of diffusible repulsive molecules [49, 52, 62]. Dscam, Ephrin and Eph are molecules that induces contact-mediated repulsion, which may also act at a long range through the filopodia [29, 32, 39, 43, 60]. Netrin, Semaphorin and Wnt are also known to act as diffusible attractive molecules. Interestingly, Netrin has been proposed to switch between attractant and repellent depending on the receptor configuration and/or ligand concentration [49]. Our study indicates the need of identifying the molecular basis of medium-range repulsion and long-range attraction that act on the arrangement of the columns in the fly brain.

The other issue that needs to be addressed is the difference in working distance between repulsive and attractive guidance molecules. Since the binding of the diffusible ligand to its receptor induces its intracellular uptake and the removal of the extracellular ligand, the receptors of the guidance molecules may play a role in determining their working distance [64]. Future studies are needed to identify multiple guidance molecules and their receptors that are involved in the process of column arrangement.

While we only focused on three cell types, R7, R8, and Mi1, in this study, there are many other cells that contribute to the formation of the columns [51]. While the column formation is initiated by these three neurons during the larval stage, other neurons and glial cells are involved in later processes during pupal development [50, 57]. As the number of cells that constitute the column increases during pupal development, the brain becomes larger. In later stages of pupal development, the columnar structure, which is regarded as a two-dimensional in this study, expands into a three-dimensional structure. Eventually, the mature column becomes very complex, containing as many as one hundred cell types in the adult brain. Unfortunately, the R7 marker used in this study, Fas2, does not work properly as a column marker at a later stage. Although we have a complete picture of the cellular components of the columns at the adult stage based on the magnificent EM reconstruction studies [13, 51, 63], the overall developmental processes of column formation are only poorly understood. Therefore, our strategy in this study is to focus on the early process of column formation and the core columnar neurons, R7, R8, and Mi1 for simplicity.

### Physiological Significance of Column Arrangement

The compound eye of *Drosophila* consists of regularly arranged hexagonal unit eyes, ommatidia, which correspond to columns in the visual center of the brain and are connected by topographic projections of photoreceptor axons. The neurites of columnar neurons are responsible for the transfer of information between neighboring columns, so the arrangement of ommatidia and columns is thought to be directly related to the information processing between neighboring columns. A regular arrangement of ommatidia and columns is considered important for information processing in the visual system. For example, direction selectivity of motion-detection cells is one of the key properties in the visual system. There are four subtypes of motion-detection cells in the fly visual center, each of which responds specifically to movement in four different directions: up, down, left, and right [27, 34, 51]. They each extend their dendrites in four different directions and are thought to be optimized for a tetragonal arrangement of columns to receive visual information from neighboring columns. On the other hand, two additional subtypes have been shown from recent studies to respond to movement in six different directions, suggesting that they may in fact be optimized for a hexagonal arrangement [25]. Since the six different subtypes are not evenly distributed in the brain, the tile patterns that are intermediate between hexagonal and tetragonal may have a benefit. Thus, although column arrangement is expected to have important significance in visual information processing, the physiological significance of column arrangement remain elusive. Careful comparison of the geometrical patterning of columns and the physiological function of neural circuits should clarify this problem.

### Concluding Remarks

In a biological aspect, we focused on the early process of column formation and the core columnar neurons in the fly brain. We developed a quantitative method to analyze the symmetric distribution patterns of cellular units such as columns. Finally, we developed the ARA model, which is an important achievement in mathematical biology because it provides a way to relate the unconventional physical potential to biological molecules such as adhesion, repulsion and attraction. By choosing appropriate parameters, the ARA model could reproduce the changes in column arrangement upon decreasing and increasing the expression level of Ncad. While we need to further expand our study in the future, the current study provides a mathematical basis for studying a wide range of tile patterns found in biological and non-biological systems.
